# Positive feedback and synchronized bursts in neuronal cultures

**DOI:** 10.1371/journal.pone.0187276

**Published:** 2017-11-01

**Authors:** Yu-Ting Huang, Yu-Lin Chang, Chun-Chung Chen, Pik-Yin Lai, C. K. Chan

**Affiliations:** 1 Dept. of Physics and Center for Complex Systems, National Central University, Chungli, Taiwan 320, ROC; 2 Institute of Physics, Academia Sinica, Nankang, Taipei, Taiwan 115, ROC; University of California San Diego, UNITED STATES

## Abstract

Synchronized bursts (SBs) with complex structures are common in neuronal cultures. Although the phenomenon of SBs has been discovered for a long time, its origin is still unclear. Here, we investigate the properties of these SBs in cultures grown on a multi-electrode array. We find that structures of these SBs are related to the different developmental stages of the cultures and these structures can be modified by changing the magnesium concentration in the culture medium; indicating that synaptic mechanism is involved in the generation of SBs. A model based on short term synaptic plasticity (STSP), recurrent connections and astrocytic recycling of neurotransmitters has been developed successfully to understand the observed structures of SBs in experiments. A phase diagram obtained from this model shows that networks exhibiting SBs are in a complex oscillatory state due to large enough positive feedback provided by synaptic facilitation and recurrent connections. In this model, while STSP controls the fast oscillations (∼ 100 ms) within a SB, the astrocytic recycling determines the slow time scale (∼10 s) of inter-burst intervals. Our study suggests that glia-neuron interactions can be important in the understanding of the complex dynamics of neuronal networks.

## Introduction

Synchronized bursts (SBs) are common in our brains. They originate from the collective dynamics of neurons in the neural networks. These bursts can be related to the normal functioning of the brain or to some pathological states such as epilepsy. SBs have been suggested as crucial in visual system development [1], sensory processing [2], neuronal information transmission [3], learning and memory [4]. Neuronal cultures grown on top of a multi-electrode array (MEA) have become a standard experimental platform for the study of SBs due to the rich patterns which can be observed in different cultured conditions [5]. Some studies try to understand SBs by manipulating culture conditions such as density [6, 7], size [8], developmental stages [9, 10] or pharmacological conditions [11]. Other studies analyze the dynamical properties of SBs such as self-regulated complexity [12] or dynamic attractors [13]. This rich repository of dynamics are considered as computational capabilities of living neural networks. For example, Ben-Jacob *et al*. studied these SBs for encoded information [14], complexities and even memories capabilities [12]. The goal of these kind of works is to understand the mechanisms of SBs and ultimately to construct a living neural chip [15] with useful functions. Unfortunately, very little success has been achieved towards this goal despite of the extensive efforts in the last two decades.

Modeling neuronal network activities to match experimental observations is a method to understand the mechanism of SBs. Numerous models have been proposed to explain the generation of SBs [16, 17]. One of the difficulties for the modeling of SBs is that the dynamics of the SBs can vary widely depending on the conditions of the cultures. It is known that SBs are controlled by synaptic transmission [18] and occur only when there are enough connections [19] in the network. Intuitively, minimal recurrent connections are needed for the re-excitation (positive feedback) of the network to maintain a burst while synaptic mechanism should determine the detailed dynamics of a SB. The diverse patterns of SBs might be generated by simple dynamical systems, which with different amount of positive feedback, can lead to complex behaviors. For the synaptic mechanism, usually only the interaction between the pre- and post-synaptic cells are considered such as the short term synaptic plasticity (STSP) model [20, 21]. However, recently, there are mounting evidence that glia cells [22–25] might also play a role in regulating synaptic dynamics.

In this article, we report our investigation by experiments and modeling of a neuronal network grown on a MEA to understand the origin of these SBs. Specifically, we are able to produce a special form of SBs similar to reverberations [26] or superbusrts [5, 27, 28] by controlling the neuron densities in the cultures. The dynamics of the SBs are characterized by the firing-rate-time-histogram (FRTH); with the shapes of these FRTH corresponding consistently well to different stages of development of the network. A mean field model based on a recurrent connection *J* and short term synaptic plasticity (STSP) is constructed to reproduce these measured FRTHs. We find that the STSP mechanism is able to reproduce generic features of the FRTHs only when an additional glial recycling of neurotransmitters mechanism characterized by the baseline level of the available neurotransmitter (*X*_0_) is included. The result of astrocytic glutamate transporter blocker (dihydrokainate) treatment experiment confirms that the additional recycling of neurotransmitters mechanism related to astrocyte. A phase diagram in terms of *J* and *X*_0_ shows that networks exhibiting SBs are in a complex oscillatory state due to the positive feedback provided by synaptic facilitation and recurrent network structure. Our finding suggests that, for the types of SBs studied in our experiments, the occurrence of SBs signals that there are too many connections in the network. In this model, while STSP controls the fast oscillations (∼ 100 ms) within a SB, the astrocytic recycling determines the slow time scale (∼10 s) of inter-burst intervals. Our study suggests that glia-neuron interactions can be important in the understanding of the complex dynamics of neuronal networks.

## Materials and methods

The cell culture development method and the measurement technique of using a multi-electrode array system employed in this study are similar to Ref. [5] and Ref. [14]. These are standard methods to generate and study the synchronized bursts events in cortical cultures in current literatures. The novelty involved in this study is the extension of a well known synaptic model (TM model, see below) to include the effects of glial cells through their recycling of glutamate from the synapses to understand the mechanism for the generation of the bursting events. Two pharmacological chemicals are used in the experiments. While [Mg^2+^] is used to change the synaptic strength of the connections in the cultures to produce different SBs, dihydrokainate (DHK), a glia glutamate transporter blocker, is used to test the validity of our extended TM model.

### Cell culture

**Fig 1:**
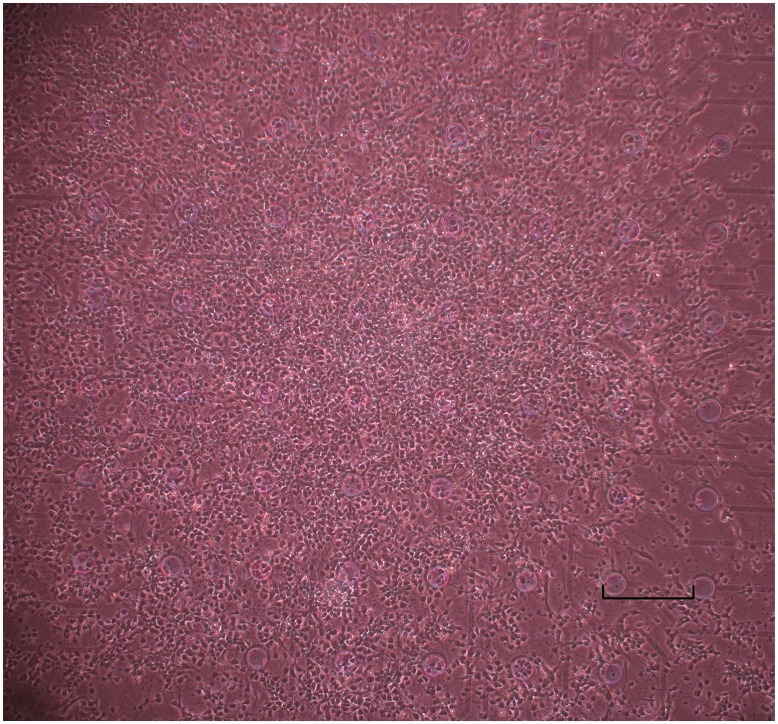
Phase contrast image of a typical culture on MEA at DIV 2. Distance between electrodes (visible in the lower right corner) is 200 *μm*.

Neuronal cultures grown on top of multi-electrode arrays (MEA) are used in our experiments. For cultures preparation [19], cortex are extracted from embryonic day 17 (E17) Wistar rat embryos. The cortical tissues are digested 15 min by 0.125% trypsin under 37°C and gentle triturated by a fire-polished Pasteur pipette to isolate cells. A small drop (5 *μ*L) of cell suspension is added on the MEA (MEA60-200-ITO, Qwane Biosciences) that has been pre-treated with 0.1% Polyethylenimine, yielding a density of 3.5 × 10^3^ cells/ mm^2^ as shown in Fig 1. Both the cells types and density reported here are chosen to be close to those reported in the literatures for similar studies [5, 13]. The MEAs are then filled with 1 mL culture medium (DMEM with 5% FBS, 5% HS and 1% penicillin/streptomycin) 30 min after the seeding. Samples are incubated at 37°C with 5% CO_2_ and 95% air. Note that no bubbling of CO_2_ into the culture medium is needed. Half of the medium is changed twice a week. All the samples from animals were prepared according guidelines from the Guide for the Care and Use of laboratory animals. The animal care and used is approved by Academia Sinica IACUC (Protocol:12-12-475).

### Signal recordings

In our MEA system, firing activities from the cultures are recorded extracellularly by 60 electrodes. The electrodes are made of transparent ITO with 40 *μ*m diameter and 200 *μ*m spacing; arranged in a 8 × 8 square grid without the 4 corners. Signals are recorded by a MEA 1060-Inv-BC (Multi Channel systems) with 1100X amplification at a sampling rate of 20 kHz. Firing activities of the cultures are recorded using MC_RACK software (Multi Channel Systems). The samples are placed in a chamber which is maintained at 37°C and filled with 5% CO_2_ and 95% air as a recording condition. Before recording, samples are kept in the recording condition for 10 minutes for adaptation. The recording session for all the experiments are 10 minutes. Spontaneous firing (baseline) are recorded with samples in culture medium [19] from 6 to 22 DIV. The results reported here are obtained from 18 samples from 7 dissections.

### Burst detection

**Fig 2:**
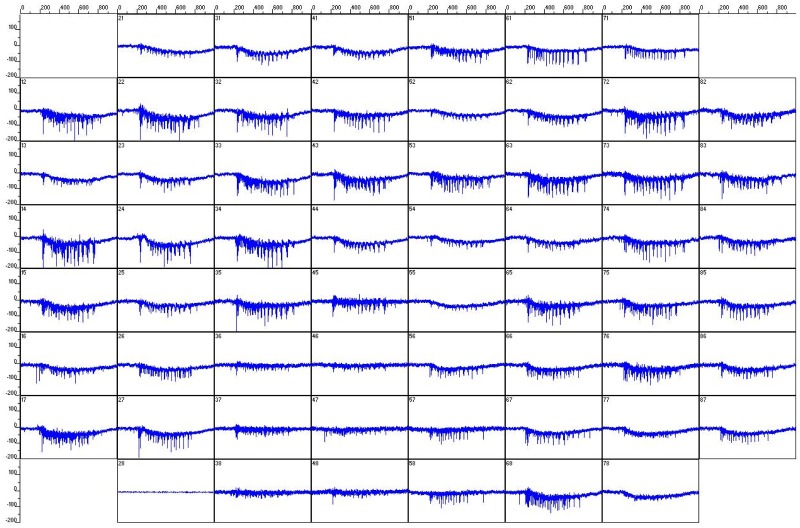
A typical signal recording from a culture at 10 DIV obtained from MEA showing synchronization across the 60 channels. The scales of each window are ±200 *μV* (y-axis) and 1 second (x-axis).

Fig 2 shows a snapshot of a 1 second recording (local field potentials) of all the 60 channels during a SB. It can be seen that the firing patterns in each channel looks similar, i.e., the synchronized firing across all the channels. Low frequency components such as drift of baseline local field potential of the data are removed by a high-pass filter at 200 Hz. Spikes are detected from the filtered data by the threshold method with five standard deviations of the data channel by channel. A SB is defined [29] as a sustained (longer than 100 ms) high firing rate (higher than 2000 Hz) event across more than 20 electrodes in the MEA. Firing activities will be excluded (not classified as SB) if the MEA can only record the activities from less than 20 channels. This lack of synchronization across most of the MEA may originate from the condition of recording chips or the condition of cultures.

**Fig 3:**
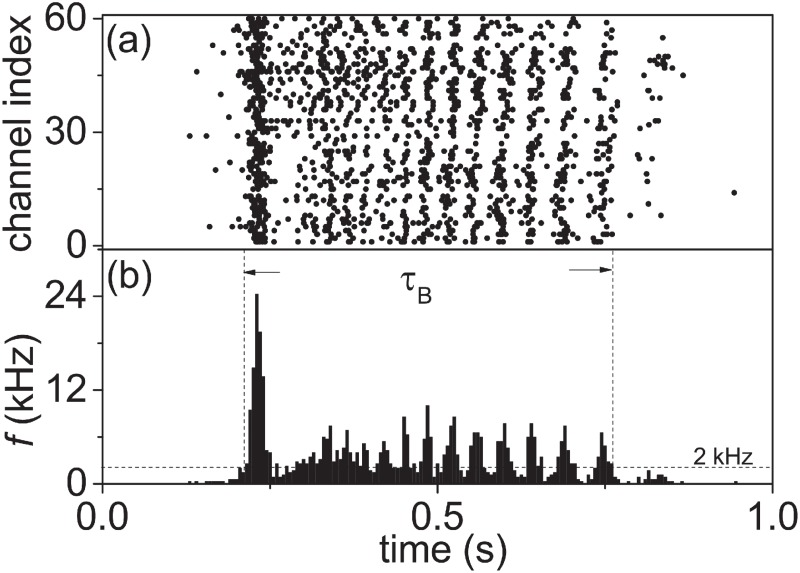
Raster plot and its firing-rate-time-histogram (FRTH) of a synchronized burst. a) Raster plots of spiking events (a dot) in the 60 channels of the MEA as a function of time. b) FRTH in a 5 ms time window constructed from a) together with the definition of burst duration (*τ*_*B*_). The 2 kHz threshold used for the detection of burst is also shown. The same data set of Fig 2 is used here.

**Fig 4:**
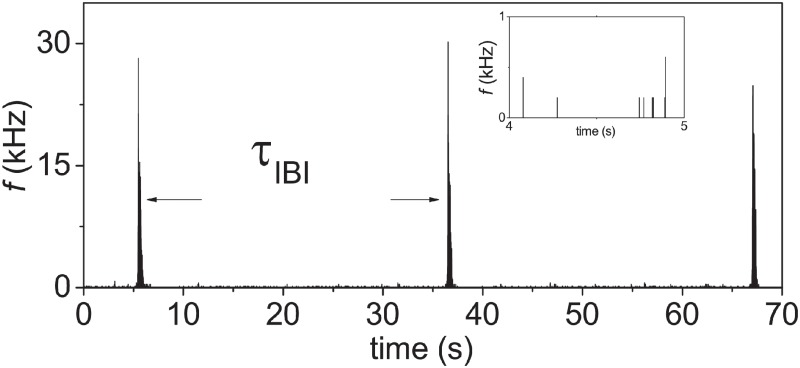
The phenomenon of SBs at a larger time scale. Three SBs are shown together with the definition of inter-burst interval (*τ*_*IBI*_) for a sample at 10 DIV. The inset shows the much smaller firing events outside of the SBs. The same data set of Fig 2 is used here.

After spike detection, a raster plot of detected spikes can be constructed from Fig 2 as shown in Fig 3(a). A remarkable feature of the raster plot is that the spikes are not distributed evenly during the burst. The spikes within the burst seem to be clustered into sub-bursts. To describe these sub-bursts clearly and quantify SBs, a firing-rate-time-histogram (FRTH) (Fig 3(b)) of a SB can be constructed from the raster plot by calculating the firing rate with a non-overlapped 5 ms window from all the 60 channels. The definition of burst duration *τ*_*B*_ is also shown in the figure. In our experiments, we find that more than 75% and 95% of the spikes recorded by the MEA are distributed inside SBs for samples in early and late DIVs respectively as shown in the Fig 4. In between two consecutive SBs, isolated spikes and a few of small local burst events, can be found as shown in the inset of Fig 4. Therefore, these SBs carry most of the information about the state of the culture. For two detected SBs with inter-burst interval (*τ*_*IBI*_) less than 1 second apart, they will be defined as belonging to the same SB. In other words, for our reported SBs below, the minimum of *τ*_*IBI*_ has to be longer than 1 second. A SB in our experiments typically has a burst duration (*τ*_*B*_) in the order of hundreds of milliseconds.

### Pharmacological tests

The extra-cellular magnesium concentration [Mg^2+^] can alter the synaptic strength of the cultures [30]; we will make use of this property to alter the state of the cultures. Since the [Mg^2+^] in the culture medium is fixed, a common method for recording with various [Mg^2+^] is to replace the culture medium by a balanced salt solution (BSS) with various amount of [Mg^2+^] [31]. The culture medium of the samples are replaced by the buffer solution 10 minutes before the recording for stabilization. The BBS contains (in mM): 130 NaCl, 5.4 KCl, 1.8 CaCl_2_, 5.5 Glucose, 20 HEPES with 0.0mM or 0.8 mM MgCl_2_ [19]. The samples are recorded for 10 minutes at 37°C without supplying CO_2_ as CO_2_ is not needed by the cultures under buffer solution. Note that the culture medium contained 0.8 mM [Mg^2+^]. The results in [Mg^2+^] treatments are observed in 12 samples from 6 dissections.

To test the validity of our TMX model (a glia involved model to be described below), we have performed experiments with a glia glutamate transporter blocker; namely dihydrokainate (DHK, Sigma D1064). Experiments with DHK are performed with cultures kept in culture medium. Drops of 10 mM DHK stock solution are added into the culture medium (1 mL) and gentled pipetted to achieve the final DHK concentrations from 100 to 500 mM with intervals of 100 mM. Recording is then started after a 2 minute break for stabilization. The results of DHK reported below are observed in 3 samples from 3 dissections. The samples are recorded for 10 minutes at 37°C with 5% CO_2_ and 95% air.

### Statistical analysis

Measurements of the experiments are reported here by their mean values with one standard deviation being shown as error bar. For each property, we calculate the mean value of each sample first and then take average over all the 18 samples. The mean burst duration $\bar{\tau_{B}}$ is defined as:

$$\begin{array}{c} \bar{\tau_{B}}=\frac{1}{N}\sum_{i=1}^{N}(\frac{1}{m}\sum_{j=1}^{m}\tau_{B}^{m})=\frac{1}{N}\sum_{i=1}^{N}(\tau_{B}^{i}) \end{array}$$ (1)

and the standard deviation *σ*(*τ*_*B*_) is defined as:

$$\begin{array}{c} \sigma \left(\tau_{B}\right)=\sqrt{\frac{1}{N-1}\sum_{i=1}^{N}{\left(\tau_{B}^{i}-\bar{\tau_{B}}\right)}^{2}} \end{array}$$ (2)

where the $\tau_{{}_{B}}^{m}$ is the m-th burst duration of a sample. Since we are only using these quantities to characterize our data for the purpose of simple comparison with our simulation model, we do not fit or test our data for any known statistical distributions. Therefore, our data can only be used for trend comparison with our simulation results; not for the detailed testing of our purposed mathematical model.

However, for the effects of [Mg^2+^] on the cultures, we are using the p-value from the single tail t-test to show the significant difference in our data. The p-value of [Mg^2+^] treatment on old samples are calculated from 9 samples which include 395 and 967 bursts in 0.8 mM and 0 mM [Mg^2+^]. If the p-value is smaller than 5% or 0.5%, the symbols * or *** will be indicated respectively. Note that the p-value is calculate from the mean and standard deviation of the pair groups and that the data is not tested for normal distribution.

### TM model

During a SB, most of neurons in the cultures are firing synchronously; suggesting that SB can be treated as a mean-field phenomenon; namely the dynamics of the whole network is similar to the dynamics of a single cell [32]. In fact, under this synchronous condition, this view that dynamics of the network can be obtained from the information of a single cell is also supported by a recent report [33]. With this picture, the mechanism of these SBs can be understood through the modeling of the mean firing rate *E*(*t*) of a single cell. Below, we will make use of the Tsodyks-Markram (TM) [34] model of STSP for this purpose and then extend it with the effects of glia to understand the mechanism for the generation of SB in cultures.

In the TM model, *E*(*t*) of a recurrent network receiving a global inhibition *I*_0_ is governed by (Supplementary Materials of [34]):

$$\begin{array}{c} \frac{dE}{dt}=\frac{1}{\tau }[-E+\alpha \ln(1+e^{\frac{JuxE+I_{0}}{\alpha }})] \end{array}$$ (3)

where *α* is the threshold of the gain function and *u* is the release probability of the available neurotransmitter fraction (*x*). Note that the positive feedback (*JuxE*) contains both the structural (*J*) and synaptic factors (*ux*). In the TM model, the dynamics of the depression and facilitation in the synaptic factors are implemented as:

$$\begin{array}{c} \frac{dx}{dt}=\frac{\chi_{0}-x}{\tau_{D}}-uxE \end{array}$$ (4)

$$\begin{array}{c} \frac{du}{dt}=\frac{U-u}{\tau_{F}}+U\left(1-u\right)E. \end{array}$$ (5)

where *χ*_0_ and *U* are the baseline level of *x* and *u* respectively. The time scales are: *τ* ∼ 10, *τ*_*D*_ ∼ 100 and *τ*_*F*_ ∼ 1000 ms. In this TM model, the synaptic dynamics is not affect by the presence of glia. In the next section, we will show that this TM model is insufficient to explain our experimental observation and the effects of glia must be included. We will use an extension of this TM model to include the effects of glia on the synaptic dynamics to understand our experimental observations.

## Results of experiments

### Properties of synchronized bursts

**Fig 5:**
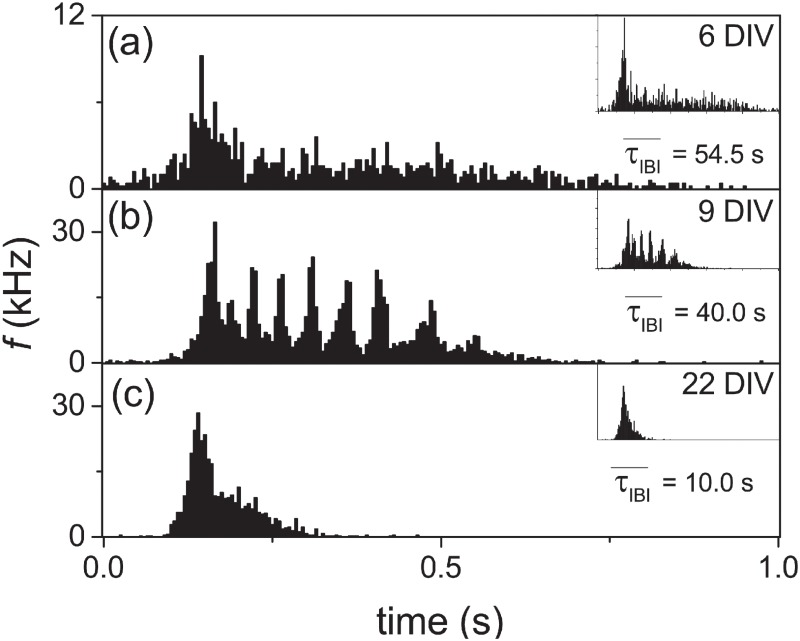
Three different types of SBs at different DIVs. Structures of FRTH at (a) 6 DIV, (b) 9 DIV and (c) 22 DIV. The mean inter-burst interval ($\bar{\tau_{{}_{IBI}}}$) are also shown in the figures. The insets are the FRTHs of another SB in the same recording.

Similar to other experiments [5], in our experiments, SBs can be observed from the cultures around 6 days after the seeding (6 DIV) and characterized by FRTHs. A remarkable feature of the FRTHs observed is that FRTHs from different SBs measured within 30 minutes from the same sample all have similar features. Fig 5 shows two measured FRTHs from the same sample within 10 minutes as a function of DIV with one of them being shown in the insets. The similarity of shapes of these FRTHs suggests that the network mechanism responsible for the SB is just repeating itself during different SBs. Thus, a FRTH constructed from anyone of the SBs can be used to represent the state of the network.

The measured FRTHs can be classified into three types based on the shapes of the FRTHs as shown in Fig 5. In the first type (< 8 DIV), SBs can be observed with a low firing rate and there is only one single peak in the FRTH. At this stage, about 75% of the total detected spikes are contained in these SBs. At the second type (9< DIV < 15), sub-peaks can be observed within the FRTH. For example, on 9 DIV, seven sub-bursts can be seen in Fig 5b. At later DIVs, in the third type (DIV> 15), the sub-peaks disappear and the SB is then consisted only of a single peak similar to that of the first stage but at a higher firing rate and short *τ*_*IBI*_. At this stage, more than 95% of the total detected spikes are contained in the SBs. It suggests that the system is more synchronized than the first stage.

**Fig 6:**
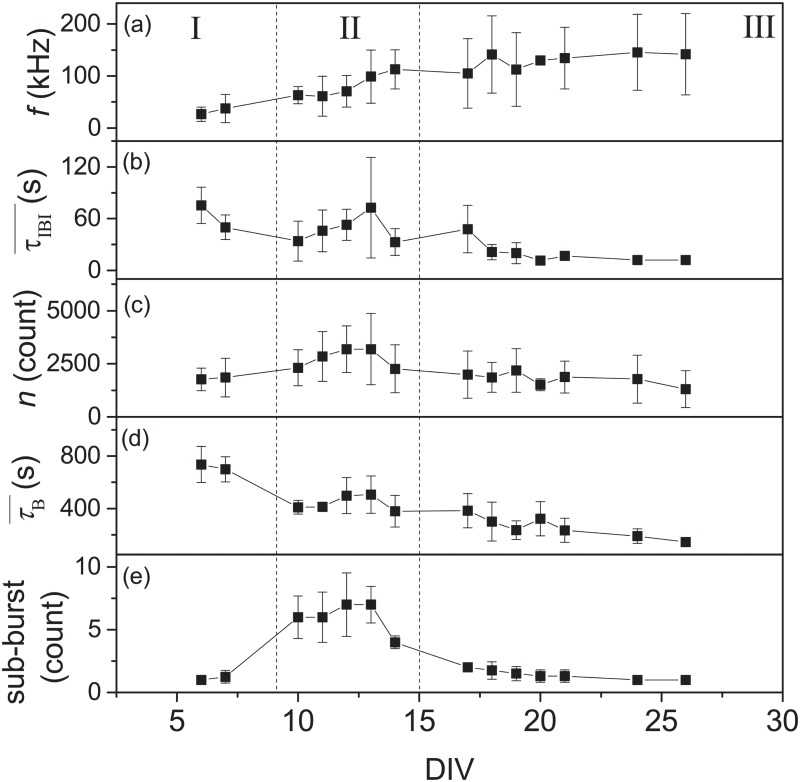
Statistical properties of SBs at different DIVs. Measured quantities of SBs as a function of DIV (N = 18): a) Mean firing rate (*f*) within 10 min. b) Mean inter-burst interval ($\bar{\tau_{{}_{IBI}}}$). c) Average number of spikes within SBs (*n*). d) Mean burst duration $\bar{\tau_{B}}$. e) Mean number of spikes within a burst. The error bars are one standard deviation from 18 samples. The classification of three types of SBs are also shown as I, II and III. Note that for a), the firing rate is computed from all the detected spikes not just limited to those within a SB.

These three types of SBs can also be characterized quantitatively by their firing rates (*f*), inter-burst interval (*τ*_*IBI*_), burst duration (*τ*_*B*_), the number of spikes within a burst (*n*) and the number of sub-burst. The DIV dependence of these various quantities are shown in Fig 6. The figure shows that *f* increases monotonically with DIV while $\bar{\tau_{{}_{IBI}}}$, $\bar{\tau_{B}}$ and *n* increase with DIV only during the occurrence of the second type of SBs. At later DIVs, these quantities decrease gradually. During the occurrence of the second type of SB, there are several sub-bursts within on SB as shown in the Fig 6e. These sub-bursts are typically with a inter-burst interval in tens of milliseconds. In contrast, the $\bar{\tau_{{}_{IBI}}}$ are in the order of tens of seconds.

### Effects of [Mg^2+^] on SB

**Fig 7:**
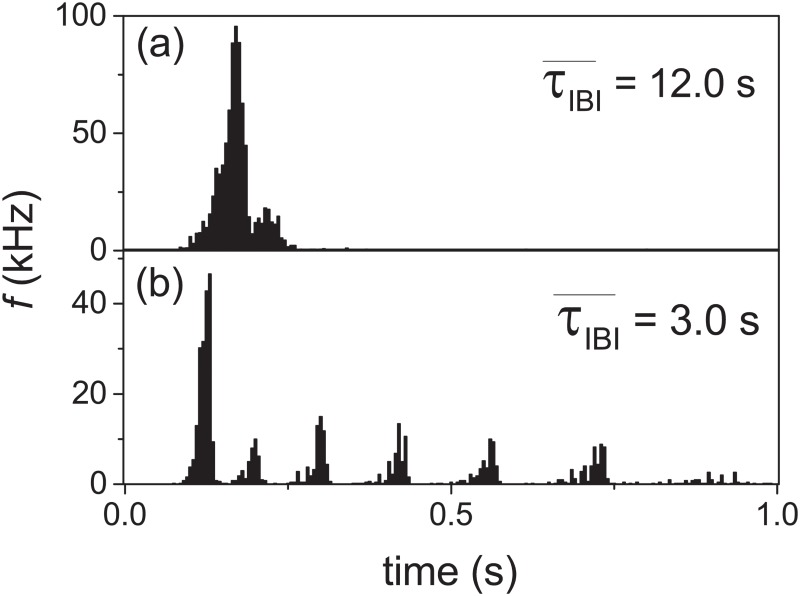
Induction of sub-bursts by removal of [Mg^2+^]. Effect of [Mg^2+^] on the FRTH for a sample at 34 DIV: (a) In culture medium with 0.8 mM [Mg^2+^]. (b) In BSS with 0 mM [Mg^2+^].

**Fig 8:**
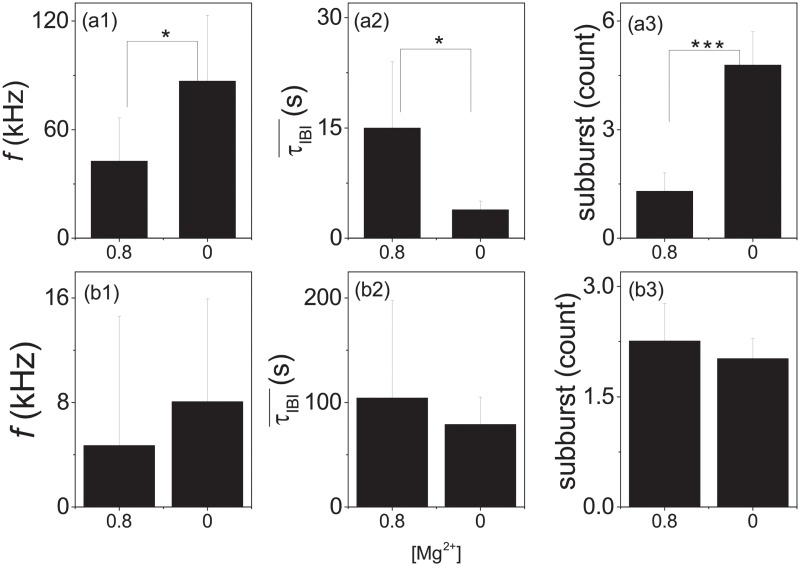
Statistical analysis of the effects of [Mg^2+^] on properties of SBs for early and late DIVs. The firing rate (*f*), inter-burst interval ($\bar{\tau_{{}_{IBI}}}$) and the number of sub-burst measured with different [Mg^2+^] concentrations for old (a1:a3, DIV>15, N = 9, bursts = 395 and 967 in 0.8 mM and 0 mM [Mg^2+^]) and young (b1:b3, DIV<15, n = 3) cultures. P<0.05 (*); P<0.005 (***).

Fig 5 shows that different developmental stages of the culture can be represented by FRTHs with different characteristics. To test the effects of synaptic mechanism on the features of measured FRTHs, we have performed experiments with reduced extracellular magnesium concentrations ([Mg^2+^]) which can modify the efficacy of synaptic connections through the blocking of the NMDA receptors. Fig 7 shows the effects of [Mg^2+^] on the structure of FRTHs. It shows that sub-bursts within the SB can be induced by decreasing the [Mg^2+^] from the normal value of 0.8 to 0 mM. The FRTHs shown in Fig 7 have similar properties of those shown in Fig 5; namely FRTHs constructed from different SBs share similar features. The result of Fig 7 suggests that the features in the measured FRTHs are controlled jointly by the network structure and synaptic mechanism. Fig 8 shows the *f*, the $\bar{\tau_{{}_{IBI}}}$, and the sub-burst number in the old and the young samples under 0.8 mM and 0 mM [Mg^2+^] concentration. Interestingly, the number of sub-burst can be induced significantly from one to six in the old samples but not the young ones.

### Effects of DHK on SB

**Fig 9:**
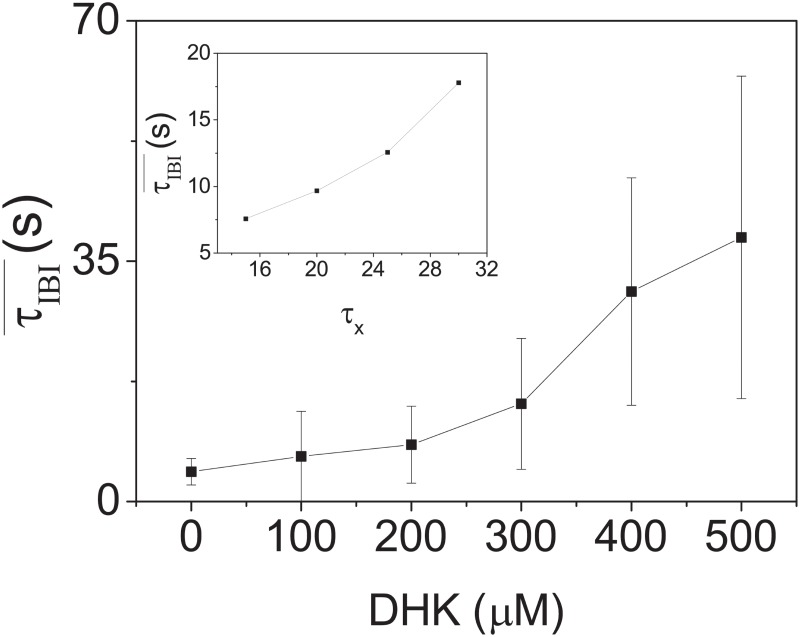
Measurement of $\bar{\tau_{{}_{IBI}}}$ as a function of concentration of DHK for cultures at 33 to 34 DIVs (N = 3). The error bars are one standard deviation from 3 samples. The inset shows a comparison to simulation results from the TMX model described in the text. Note that *τ*_*x*_ controls the recycling time of neurotransmitters; playing similar role of DHK. Parameters for the TMX model are *X*_0_ = 0.95, *J* = 5.8, *U* = 0.3, *I*_0_ = −1.3, *β* = 0.01, *τ* = 0.013 s, *τ*_*D*_ = 0.15 s, *τ*_*F*_ = 1.5 s and *α* = 1.5.

The effects of glia on the properties SB are also investigated by the use of DHK; an astrocytic glutamate transporter (GLT-1) blocker [35]. It is known that astrocytes will convert the uptaken glutamate from synapses to glutamine and then transfer the glutamine back to a presynaptic neuron for further synaptic releases. It is possible that this recycling of glutamate mechanism can have an impact on the dynamics on the generation of SBs. Intuitively, a partial blocking of this recycling mechanism will slow down the generation of SBs because it will take longer time for there to be enough glutamate in the presynaptic cell to generate the next SB. To test this idea, experiments are performed with DHK. Fig 9 is a measurement of $\bar{\tau_{{}_{IBI}}}$ as a function of DHK concentration. It shows clearly that the generation of SBs has indeed been slowed down by an increase in DHK concentration in the culture.

## Results of simulation model

### Extension of TM model

In the original TM model (*χ*_0_ = 1), with a big enough positive feedback (*J* and *U*), the TM model can produce oscillations [36]. However, the time scales of these oscillation are too short to describe the time scale of the SBs observed in the experiments. These time scales are more similar to the time scales of the sub-bursts shown in Fig 5 but these oscillations do not stop as the sub-bursts observed in experiments. If these oscillations induced by positive feedback in the TM model are indeed the sub-bursts seen in the experiments, we need to modulate the amount of the positive feedback to stop the sub-bursts. One could control either *x* or *u* through modulating their base values *χ*_0_ or *U* respectively. Since *U* controls the sub-burst oscillations and the recycling of *x* will be affected due to repeated firings [37], we choose to control *x* by introducing a time dependent *χ*_0_ which is modeled as:

$$\begin{array}{c} \frac{d\chi_{0}}{dt}=\frac{X_{0}-\chi_{0}}{\tau_{X}}-\beta E \end{array}$$ (6)

for some baseline constant *X*_0_, time constant *τ*_*X*_(>> *τ*_*D*_) and a fatigue rate constant *β*. Here one expects the fatigue rate is an even slower process with *β* << 1/*τ*_*X*_, representing that prolong firing would render significant portion of the neurotransmitters not be available [32]. We will refer to this extension as the TMX model. Note that *χ*_0_ is similar to the “super-inactive state” used by Volman [38] to model reverberations in cultures.

### Properties of TMX model

**Fig 10:**
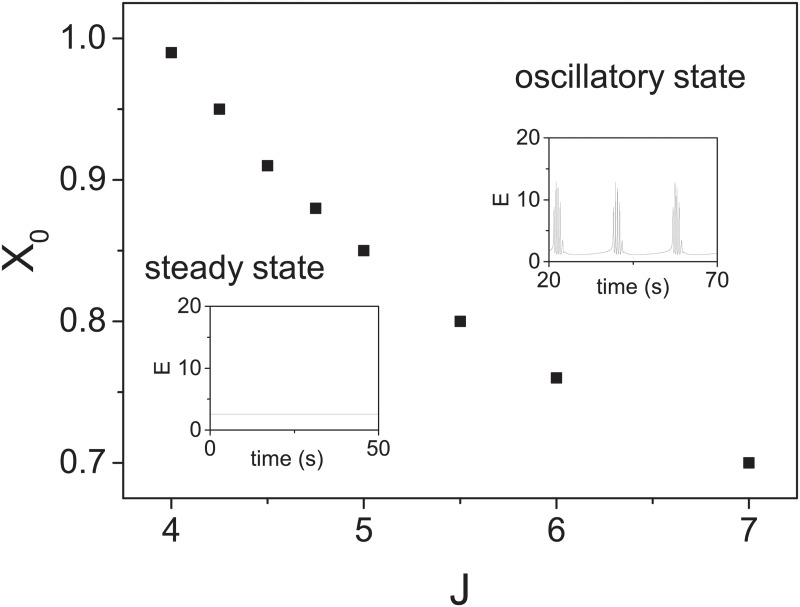
Phase diagram of oscillation state of the TMX model. Minimal *X*_0_ for eliciting an oscillatory firing for a given value of *J* for *U* = 0.3 and *τ*_*D*_ = 0.15 s. Other parameters are *τ*_*X*_ = 20 s, *I*_0_ = −1.3, *β* = 0.01, *τ* = 0.013 s, *τ*_*F*_ = 1.5 s and *α* = 1.5. The insets show the characteristic of *E*(*t*) (in Hz) in the steady and the oscillatory states.

The TMX model has at least two interesting states. The first one is a low firing rate (*E* ∼ *O*(1)) steady state and the other is a periodic state with *E*(*t*) showing sub-burst oscillations. This latter state is just the persistent oscillatory state of the TM model now modulated by a time dependent *χ*_0_. One can regard the low firing rate state as the cultures in early DIV with little activities and the periodic *E*(*t*) as the system at later DIV with SBs. Fig 10 shows a phase diagram for these states in terms of *J* and *X*_0_. Note that the system will be in the periodic state only when either *X*_0_ or *J* (positive feedback) is large enough.

### Comparison with DIV dependence

**Fig 11:**
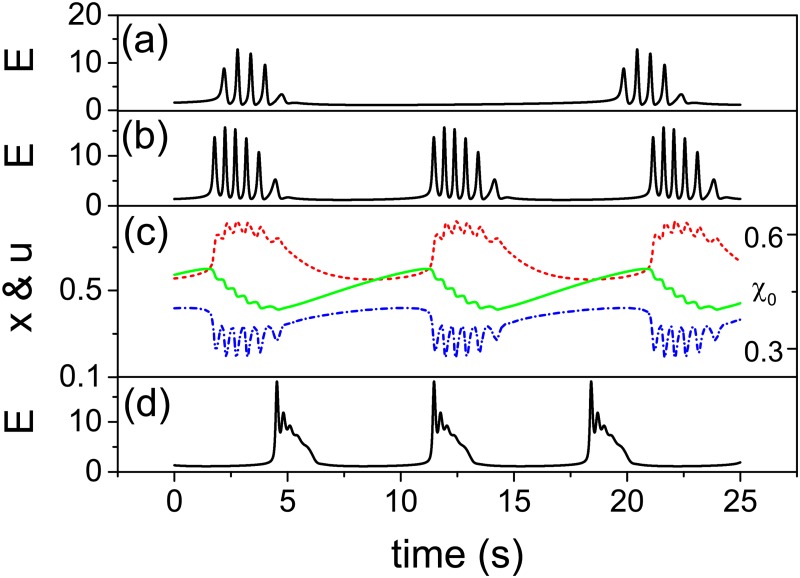
Time courses of *E*(*t*) (in Hz) of the TMX model to mimic the FRTH at different DIV. (a) *J* = 4.8, *τ*_*D*_ = 0.2 s and *U* = 0.28. (b) *J* = 5.8, *τ*_*D*_ = 0.15 s and *U* = 0.3. (d) *J* = 6.8, *τ*_*D*_ = 0.1 s and *U* = 0.32. (c) Corresponding time courses of *x* (blue dash-dot), *u* (red dot) and *χ*_0_ (green) for time course (b). Other parameters are the same as Fig 10.

To demonstrate that this TMX model can reproduce essential features of the FRTHs observed in experiments, we have shown in Fig 11 different forms of *E*(*t*) produced from the TMX model to mimic the effects of DIV. The figure shows that there are sub-bursts within these *E*(*t*) and the number of sub-bursts decreases; similar late DIV FRTH shown in Fig 5. In addition, the *τ*_*IBI*_ also decreases, agreeing with the experimental results. Note that the time scale of the *E*(*t*) is comparable to that of the experiments. In order to generate Fig 11, we have assumed i) *J* and *U* increase with DIV as the cultures become mature and ii) the neurotransmitter re-cycling process becomes faster as the neuron niche improves [39]. Fig 11c are the time courses of *x*, *u* and *χ*_0_. The figure shows that the interaction between *x* and *u* generates the sub-bursts while the depletion and the recovery of *χ*_0_ controls the stop and the start of the SB. Note that when *τ*_*D*_ is small (fast recycling), the sub-bursts can get very close and eventually disappear (Fig 11d).

### Comparison with [Mg^2+^] dependence

**Fig 12:**
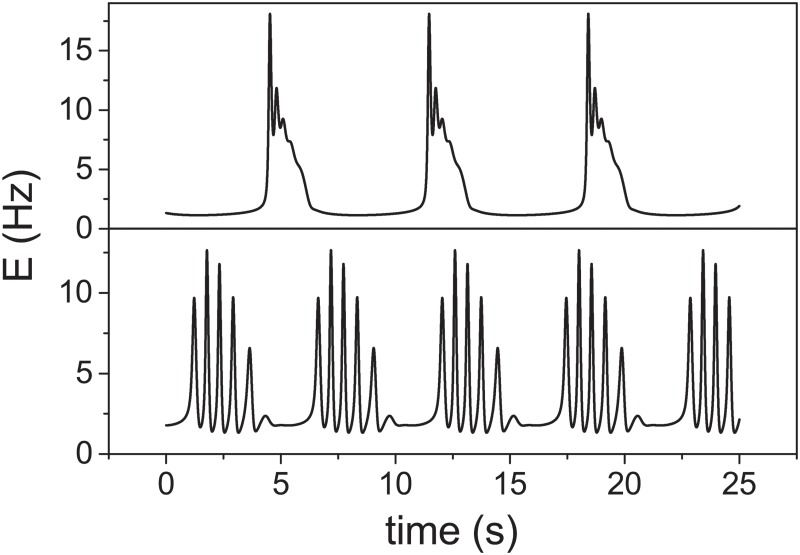
Time courses of *E*(*t*) (in Hz) of the TMX model to mimic different magnesium treatment. *E*(*t*) (in Hz) from the TMX model for situations of a) normal [Mg^2+^]: *J* = 6.8 and *τ*_*D*_ = 0.1 s and b) low [Mg^2+^]: *J* = 7.8 and *τ*_*D*_ = 0.15 s. Other parameters are *τ*_*X*_ = 20 s, *I*_0_ = −1.3, *β* = 0.01, *τ* = 0.013 s, *τ*_*F*_ = 1.5 s and *α* = 1.5.

To mimic the effects of [Mg^2+^] on FRTH (Fig 7), we note that the unblocking effect of the NMDA receptors by a decrease in [Mg^2+^] can be viewed as an increase in effective *J* which will lead to an increase in firing rate. Since an increase in firing rate will make the recycling of the neurotransmitters slower [40], the overall effects of a decrease in [Mg^2+^] will be modeled in the TMX model as an increase in *J* and an increase in *τ*_*D*_ as shown in the Fig 12. The result illustrates that sub-bursts can indeed be induced by the lengthening of *τ*_*D*_ while an increase in *J* shortens the *τ*_*IBI*_; similar to our experimental findings.

### Comparison with [DHK] dependence

The success of the TMX model requires the existence of the modulations of available neurotransmitters (glutamate) *χ*_0_. As mentioned above, a known mechanism for this modulation is the uptake and recycling of glutamate from the astrocytes surrounding the synapses. Changes of the recycling process will be reflected in the changes of *τ*_*X*_ in the TMX model. For example, when *τ*_*X*_ is increased in the TMX model, one will expect an increase in inter-burst interval $\bar{\tau_{{}_{IBI}}}$. In fact, this prediction is supported by our experiments with DHK as shown in Fig 9. The inset of Fig 9 is the corresponding simulation result from the TMX model. These results demonstrate that the TMX model is consistent with the recycling of glutamate by astrocytes.

## Discussions

Synchronized bursting has been discovered for a long time but its origin is still not clear. The echo-like activities within a SB, also known as reverberations or superbursts, are fine structures that can be observed not only in cortical cultures but also in neuronal stem cell development [27] and brain slice preparations [41]. Numerous experiments and modeling have been dedicated to study the phenomenon of SB and its mechanism [16, 17, 36, 37, 42]. However, most of these works are focused on the appearance, generation and maintenance of a SB. Only a few of them are focused on the details of the firing pattern during a SB [43]. Our experimental work focus on the details of these reverberations and the effects of synaptic mechanisms based on connectivity (effect of DIV), connection strength (effect of [Mg^2+^]) and glial mechanism (effect of [DHK]). To the best of our knowledge, this is the first time that the effects of glia is considered for the phenomenon of synchronized bursting. Presumably, our findings give a more satisfactory understanding of the basic mechanism of SB.

One of the difficulties in understanding of the phenomenon of SB is the coexistence of a fast and slow time scales. The time scale of the reverberating activities (sub-burst) within a SB is of the order of hundreds of milliseconds while the inter-burst interval is of the order to tens of seconds. To obtain a fast time scale, a fast mechanism such as the short-term synaptic plasticity needs to be considered. The TM model is a basic STSP model which has already been applied successfully in diverse phenomena such as network bursting [37], working memory [34] and computational capacities [44]. The dynamical properties of TM model have also been studied by Cortes *et* *al*. [36]. However, the TM model itself does contain a time scale as long as the inter-burst interval. Our extension of the TM model to include the glial mechanism of glutamate recycling is to introduce such a long time scale into the system.

The picture emerges from our TMX model is that the reverberations in a SB originate from too much positive feedback in the network due either to high recurrent connectivity (J) or strong synaptic connections (*X*_0_). Without the interaction with astrocytes, these sub-bursts will not stop. When modulation through glia is included, the sub-bursts will be stopped due to the depletion of neurotransmitters being transported by the astrocytes. In the TMX model, the fast and slow time scales originate from short term synaptic plasticity and glial glutamate recycling respectively. With this view, the effect of neurotransmitter recycling in the TMX model, which has not been considered before, is essential for the understanding of the mechanism of SB. However, it should be noted that Eq 6 of the TMX model which takes into account the effects of glia, is purely phenomenological. It is needed to stop the oscillations of the TM model by a depletion of available neuro-transmitters. The form of Eq 6 is only one of many possible forms. Its dependence on *E* and the empirical values of the constants *τ*_*χ*_ and *β* remained to be measured by further experiments. However, the important point is that a concrete example is given to support the long standing hypothesis that glia can take part in the regulation of the overall activity of a neural network. In this case, the involvement of glia turns a non-stopping oscillation into bursts. This idea is inspired by several papers demonstrating that astrocytes have the ability to affect the synchronization in neural system in both experimental [24, 35] and theoretical studies [25, 32, 45].

Several studies [19, 43, 46] have shown that network connectivity and synaptic depression are critical parameters to control burst appearance [17]. Our TMX model is also consistent with these findings if connections and synapses efficacy increases in the network as the culture matures [46]. Although the TMX model can capture some essential features of the dynamics of SBs observed in our experiments, the periodicity of *E*(*t*) predicted are not seen in experiments. Presumably, it is because the mean-field nature of the TMX model will work only when the system is perfect synchronized. When the culture is in the low firing state before the occurrence of SB, the dynamics of the network cannot be described by a mean-field and the firing of a particular neuron in the system can triggered a SB. In this case, we do not expect to see the periodic generation of SBs.

Finally, it should be mentioned that both our experiments and the TMX model described above are developed for a special form of synchronized bursts; although it can be commonly observed in 2D cultures [13, 27, 28]. Since these types of SBs can also be observed in an acute slice preparation from a functional brain only when the effective recurrent connections are artificially increased by the lowering of [Mg^2+^] (a non-physiological condition [47]), the existence of SBs in a neuronal system might signal that it is perhaps in a pathological state such as epilepsy which is also characterized by synchronized firing over large area of the brain. The fact that the SBs are generated spontaneously in our 2D cultures under physiological [Mg^2+^] suggests that there might already be too many connections in the network and therefore not suitable for the study of normal functions of a neuronal system.
